# Supplementary material for: A conserved population of MHC II-restricted, innate-like, commensal-reactive T cells in the gut of humans and mice
Source: Nat Commun. 2022 Dec 3;13:7472. doi: 10.1038/s41467-022-35126-3 (PMC9719512; doi:10.1038/s41467-022-35126-3)
Supplement: Supplementary file 3 — Description of Additional Supplementary Files [file 41467_2022_35126_MOESM3_ESM.pdf]

Description of Additional Supplementary Files

Supplementary Data 1: CD161hi core gene signature

List of genes upregulated in blood-derived MAIT cells, CD161hi TCRgd cells and CD161hi CD4s compared to CD8s and the respective CD161-negative population. This list was published in a paper by Fergusson et al. in 2014 (https://www.cell.com/cell-reports/fulltext/S2211-1247(14)00828-6#relatedArticles).

Supplementary Data 2: CD161hi DEG

Differential gene expression analyses between the indicated CD4 subsets.
p-values were calculated in R using a Walds test implemented in the results function from the DESeq2 package. p-values were adjusted by the Bejamini-Hochberg method.
The CD161hi gene module (human TMIC gene module) was derived from those lists by combining the lists of genes significantly (p-adjust < 0.05) upregulated in either DP or CD161hi compared to CD161- or CD161int cells

Supplementary Data 3: PCA loading

Sheets contain the loading values for the genes driving the clustering in the PCA shown in Figure 4A. Values were obtained by exporting the information stored in the 'loadings' part of the PCA-object created using the PCAtools R package.

Supplementary Data 4: GO-term analysis

Sheets contain the full lists of GO terms associated with the genes driving PC1 or 2 from the PCA shown in Figure 4A. p-values were calculated by a one-tailed version of Fisher's exact test implemented in the clusterprofiler R package and p-values were multiple hypothesis testing by the Benjamini-Hochberg method. For Display of these terms in Figure 4 and Suppl. Figure 4 redundant terms were removed using the simplify function from the enrichplot R package and the Top20 Terms were shown.

Supplementary Data 5: DN DEG

Differential gene expression analyses between the indicated murine populations. p-values were calculated in R using a Walds test implemented in the results function from the DESeq2 package. p-values were adjusted by the Bejamini-Hochberg method. The DN gene module (murine TMIC gene module) was derived from those lists by combining the lists of genes significantly (p-adjust < 0.05) upregulated in either Cbir or wt DN cells compared to CD4s cells.

Supplementary Data 6: Rhapsody mRNA targets

List of genes which expression was analysed using the BD Rhapsody. The BD Rhapsody Immune response Panel Hs was used a basis (marked in blue) and 145 additional mRNA targets (marked in black) were added. These additional targets included genes associated with tissue repair in H2M3-restricted CD8 T cells in the skin (published by Linehan et al. in 2018) as well as additional immune-related genes.
