# Supplementary material for: A conserved population of MHC II-restricted, innate-like, commensal-reactive T cells in the gut of humans and mice
Source: Nat Commun. 2022 Dec 3;13:7472. doi: 10.1038/s41467-022-35126-3 (PMC9719512; doi:10.1038/s41467-022-35126-3)
Supplement: Supplementary file 10 — Reporting Summary [file 41467_2022_35126_MOESM10_ESM.pdf]

nature portfolio

Corresponding author(s): Emily E Thornton and Paul Klenerman

Last updated by author(s): Nov 10, 2022

Reporting Summary

Nature Portfolio wishes to improve the reproducibility of the work that we publish. This form provides structure for consistency and transparency in reporting. For further information on Nature Portfolio policies, see our Editorial Policies and the Editorial Policy Checklist.

Statistics

For all statistical analyses, confirm that the following items are present in the figure legend, table legend, main text, or Methods section.

n/a	Confirmed	
[ ]	[x]	The exact sample size (*n*) for each experimental group/condition, given as a discrete number and unit of measurement
[x]	[ ]	A statement on whether measurements were taken from distinct samples or whether the same sample was measured repeatedly
[ ]	[x]	The statistical test(s) used AND whether they are one- or two-sided<br>*Only common tests should be described solely by name; describe more complex techniques in the Methods section.*
[ ]	[x]	A description of all covariates tested
[ ]	[x]	A description of any assumptions or corrections, such as tests of normality and adjustment for multiple comparisons
[ ]	[x]	A full description of the statistical parameters including central tendency (e.g. means) or other basic estimates (e.g. regression coefficient) AND variation (e.g. standard deviation) or associated estimates of uncertainty (e.g. confidence intervals)
[ ]	[x]	For null hypothesis testing, the test statistic (e.g. *F*, *t*, *r*) with confidence intervals, effect sizes, degrees of freedom and *P* value noted<br>*Give P values as exact values whenever suitable.*
[x]	[ ]	For Bayesian analysis, information on the choice of priors and Markov chain Monte Carlo settings
[x]	[ ]	For hierarchical and complex designs, identification of the appropriate level for tests and full reporting of outcomes
[ ]	[x]	Estimates of effect sizes (e.g. Cohen's *d*, Pearson's *r*), indicating how they were calculated

Our web collection on statistics for biologists contains articles on many of the points above.

Software and code

Policy information about availability of computer code

Data collection

Flow cytometry data:
FACS Diva Software V 9.1

Data analysis

Flow cytometry data:
FlowJo Software V 10.7.1 (BD)
Prism V 9(Graphpad)

Analysis of RNASeq data:
R version 4.0.3 (2020-10-10) -- "Bunny-Wunnies Freak Out"
Rstudio version 1.3.1056 "Water Lily" (5a4dee98, 2020-07-07) for macOS
Code and functions provided by the following R packages: Annotationdbi, biomaRt, DESeq2, edgeR, fgsea, GSEABase, PCAtools, vsn.

Merging of human and mouse datasets and analyses of the merged data:
R version 4.0.3 (2020-10-10) -- "Bunny-Wunnies Freak Out"
Rstudio version 1.3.1056 "Water Lily" (5a4dee98, 2020-07-07) for macOS
Code and functions provided by the following R packages: Annotationdbi, biomaRt, clusterprofiler, edgeR, fgsea, GSEABase, LIMMA, msigdbr, PCAtools, sva, vsn,

Analysis of published single cell RNAseq datasets:
R version 4.0.3 (2020-10-10) -- "Bunny-Wunnies Freak Out"
Rstudio version 1.3.1056 "Water Lily" (5a4dee98, 2020-07-07) for macOS

Code and functions provided by the following R packages: Annotationdbi, AUCell, biomaRt, qusage, Seurat.
https://github.com/saxifragus-oxf/TMIC-project
Prism V 9(Graphpad).

Analysis of Rhapsody single cell data:
7 Bridges Genomics pipeline https://www.sevenbridges.com/
SeqGeq Software V 1.7 (BD)
R version 4.0.3 (2020-10-10) -- "Bunny-Wunnies Freak Out"
Rstudio version 1.3.1056 "Water Lily" (5a4dee98, 2020-07-07) for macOS
Code and functions provided by the following R packages: Seurat.

Analysis of human TCR sequences:
SeeTCR: http://friedmanlab.weizmann.ac.il/SeeTCR/

For manuscripts utilizing custom algorithms or software that are central to the research but not yet described in published literature, software must be made available to editors and reviewers. We strongly encourage code deposition in a community repository (e.g. GitHub). See the Nature Portfolio guidelines for submitting code & software for further information.

Data

Policy information about availability of data

All manuscripts must include a data availability statement. This statement should provide the following information, where applicable:

- Accession codes, unique identifiers, or web links for publicly available datasets
- A description of any restrictions on data availability
- For clinical datasets or third party data, please ensure that the statement adheres to our policy

All RNA sequencing datasets have been made publicly available or are available via permanent links below. All datasets will be made publicly available upon publication.

Human bulk RNASequencing: https://www.ebi.ac.uk/arrayexpress/experiments/E-MTAB-11440
Mouse datasets: https://www.ebi.ac.uk/arrayexpress/experiments/E-MTAB-11397
Human Rhapsody single cell sequencing: Omnibus database GSE207159 https://www.ncbi.nlm.nih.gov/geo/query/acc.cgi?acc=GSE207159

Field-specific reporting

Please select the one below that is the best fit for your research. If you are not sure, read the appropriate sections before making your selection.

☒ Life sciences ☐ Behavioural & social sciences ☐ Ecological, evolutionary & environmental sciences

For a reference copy of the document with all sections, see nature.com/documents/nr-reporting-summary-flat.pdf

Life sciences study design

All studies must disclose on these points even when the disclosure is negative.

Sample size

Human data:
No statistical tests were performed to determine sample size. Our ethics allowed for the collection of up to 50 specimens of each sample type. In the three years leading up to this publication in total 49 normal adjacent tissue pieces ('healthy') from CRC patients, 9 biopsie samples from patients with active UC (UCEIS > 2) and 12 biopsies from patients without inflammation could be collected. In each experiment using 'healthy' samples, material from at least 5 different patients was used.
For RNA -sequencing, 800 cells per cell population from three different donors were analyzed in bulk.

Mouse date:
Sample size was computed using an estimate of colitis score between 6–8, to detect a score reduction by 50% in a treated group with a standard deviation of 30%, false positive of 0.05, and power of 0.80. Sample size was therefore calculated to be between 6–7. Where smaller changes were expected, sample sizes were increased accordingly.

Data exclusions

Human data: No datapoints were excluded.

Mouse data: No datapoints were excluded

Replication

Human data: Samples individually were obtained over three years (2018-2021). As a result, one experiment usually was done with one and never with more than three human samples. Hence, each figure depicted in the manuscript contains datapoints from at least 3 independent experiments. Since the results were consistent over this time period we consider them highly reproducible.

Mouse data: Most experiments were performed at least three times with experiments done twice indicated in the figure legend.

Randomization	Human samples: Within a given experimental setup, all samples received the same treatment and no randomization was done. For the data presented in Figure 5, human samples were allocated to the groups (healthy, UC - noninflamed and UC - inflamed) based on the clinical diagnosis.<br><br>Mouse samples: Where possible, litter mates were used in mouse experiments. Mice were given a random ear clip before genotyping. Upon genotyping, the presence of each genotype was confirmed in each cage, but mice were not formally randomized.
Blinding	Human data: For the bulk of the analyses no blinding was performed as all the samples were from the same group of patients and received identical treatment. In the experiments involving UC samples, samples were processed without knowledge about the exact diagnosis. For the analysis the experimental results were matched with the diagnostic data based on the numerical identifiers assigned to each sample by the Biobank handling the speciemens.<br><br>Mouse samples: The random ear clip given before genotyping was used throughout the experiment to identify the mice instead of genotype, effectively blinding the experimenters. All histology scoring was performed by two blinded researchers.

Reporting for specific materials, systems and methods

We require information from authors about some types of materials, experimental systems and methods used in many studies. Here, indicate whether each material, system or method listed is relevant to your study. If you are not sure if a list item applies to your research, read the appropriate section before selecting a response.

Materials & experimental systems

n/a	Involved in the study
☐	☒ Antibodies
☒	☐ Eukaryotic cell lines
☒	☐ Palaeontology and archaeology
☐	☒ Animals and other organisms
☐	☒ Human research participants
☒	☐ Clinical data
☒	☐ Dual use research of concern

Methods

n/a	Involved in the study
☒	☐ ChIP-seq
☐	☒ Flow cytometry
☒	☐ MRI-based neuroimaging

Antibodies

Antibodies used

Flow cytometry human cells:
FITC mouse anti human CCR7 (clone: G043H7) Biolegend Cat # 353215; RRID: AB_10945291, 1:100
BV570 mouse anti human CD3 (clone: UCHT1) Biolegend Cat # 300436; RRID: AB_2562124, 1:100
PE/Dazzle 594 mouse anti human CD3 (clone: UCHT1) Biolegend Cat # 980006; RRID: AB_2715768, 1:100
PerCp/Cy5.5 mouse anti human CD3 (clone: UCHT1) Biolegend Cat # 300430; RRID: AB_893299, 1:100
BV605 mouse anti human CD4 (clone: OKT4) Biolegend Cat # 317438; RRID: AB_11218995, 1:100
BV650 mouse anti human CD4 (clone: OKT4) Biolegend Cat # 317436; RRID: AB_2563050, 1:100
BV650 mouse anti human CD8 (clone: SK1) Biolegend Cat # 344730; RRID: AB_2564510, 1:100
BV421 mouse anti human CD39 (clone: A1) Biolegend Cat # 328214; RRID: AB_2564575, 1:200
PE/Dazzle594 mouse anti human CD45RA (clone: HI100) Biolegend Cat # 304145; RRID: AB_2564078, 1:100
PE/Cy7 mouse anti human CD45RO (clone: UCHL1) Biolegend Cat # 304229; RRID:AB_11203903, 1:50
BV421 mouse anti human CD56 (clone: HCD56) Biolegend Cat # 318328; RRID: AB_11218798, 1:100
BV480 mouse anti human CD56 (clone: NCAM16.2) BD Biosciences Cat # 566124; RRID: AB_2739525, 1:100
PE/Cy7 mouse anti human CD154 (clone: 24-31) Biolegend Cat # 310832; RRID: AB_2563017, 1:50
BV421 mouse anti human CD161 (clone: HP-3G10) Biolegend Cat # 339914; RRID: AB_2561421, 1:100
PE mouse anti human CD161 (clone: 191B8) Miltenyi Cat # 130-113-593, RRID: AB_2733772, 1:200
APC mouse anti human GzmB (clone: GB11) Invitrogen Cat # GRB05;RRID: AB_2536539, 1:100
Alex Fluor 700 mouse anti human/mouse GzmB (clone: QA16A02) Biolegend Cat # 372222; RRID: AB_2728389, 1:100
PE/Vio770 human anti human/mouse/rat ICOS (clone: REA192) Miltenyi Cat # 130-100-735; RRID: AB_2656924, 1:50
Alex Fluor 700 mouse anti human IFNg (clone: 4S.B3) Biolegend Cat # 506516; RRID: AB_961351, 1:100
BV711 mouse anti human IFNg (clone: 4S.B3) Biolegend Cat # 502540; RRID: AB_2563506; 1:100
BV785 mouse anti human IFNg (clone: 4S.B3) Biolegend Cat # 502542; RRID: AB_2563882, 1:100
FITC mouse anti human IFNg (clone: 45-15) Miltenyi Cat # 130-113-492; RRID: AB_2733589, 1:100
Alexa Fluor 647 mouse IgG1k (clone: MOPC-21) BD Biosciences Cat # 557732;RRID: AB_396840, 1:50
PE mouse IgG2bk (clone: 27-35) BD Biosciences Cat # 555058; RRID: AB_395678, 1:50
BV421 mouse anti human IL-17A (clone: BL168) Biolegend Cat # 512322;RRID: AB_11218604, 1:100
FITC mouse anti human IL-17A (clone: BL168) Biolegend Cat # 512304;RRID: AB_961390, 1:00
PE rat anti human IL17F (clone: eBio18F10) Invitrogen Cat # 12-7471-82; RRID: AB_1210742, 1:100
PE/Cy7 rat anti human IL17F (clone: SHLR17) Thermo Fisher Cat # 12-7169-42; RRID: AB_2572654, 1:100
PE/Cy7 mouse anti human IL18Ra (clone: H44) Biolegend Cat # 313812; RRID: AB_2800827. 1:125
APC mouse anti human IL18Ra (clone: H44) Biolegend Cat # 313814; RRID: AB_2800829, 1:100

PerCp/eFluor 710 mouse anti human IL-22 (clone: 22URTI) Thermo Fisher Cat # 46-7229-42; RRID: AB_10596639, 1:50
PE/Cy7 mouse anti human LAG3 (clone: 7H2C65) Biolegend Cat # 369208; RRID: AB_2629835, 1:100
BV785 rat anti human PD-1 (clone: 29F.1A12) Biolegend Cat # 135225; RRID: AB_2563680, 1:50
Alex Fluor 647 mouse anti human PLZF (clone: R17-809) BD Biosciences Cat # 563490;RRID: AB_2738238, 1:50
BV711 mouse anti human TCR Va7.2 (clone: 3C10) Biolegend Cat # 351732; RRID: AB_2629680, 1:100
FITC mouse anti human TCR Va7.2 (clone: 3C10) Biolegend Cat # 351704; RRID: AB_10900975, 1:50
PerCp/Cy5.5 mouse anti human TCR Va7.2 (clone: 3C10) Biolegend Cat # 351710; RRID: AB_2561954, 1:100
BV711 mouse anti human TCR Va24-Ja18 (clone: 6B11) Biolegend Cat # 342922; RRID: AB_2572068, 1:100
PerCp/Cy5.5 mouse anti human TCR Va24-Ja18 (clone: 6B11) Biolegend Cat # 342914; RRID: AB_2562455, 1:100
PerCp/Cy5.5 mouse anti human TCRgd (clone: B1) Biolegend Cat # 331224; RRID: AB_2563013, 1:100
APC/Fire 750 mouse anti human TCRgd (clone: B1) Biolegend Cat # 331228; RRID: AB_2650627, 1:100
APC mouse anti human TIGIT (clone: A15153G) Biolegend Cat # 372706; RRID: AB_2632732, 1:100
BV605 mouse anti human TIM3 (clone: F38-2E2) Biolegend Cat # 345018; RRID: AB_2563859, 1:50
PerCp/Cy5.5 mouse anti human TNF (clone: MAb11) Biolegend Cat # 502926; RRID: AB_2204081, 1:50
PE mouse anti human RoRgt (clone: Q21-559) BD Biosciences Cat # 563081; RRID: AB_2686896 , 1:50

Blocking of human cells:
ULTRA-LEAF purified mouse IgG2ak (clone: MOPC-173) Biolegend Cat # 400264; RRID: AB_11148947, 10µg/ml
ULTRA-LEAF purified mouse IgG2bk (clone: MPC-11) Biolegend Cat # 400348, 10µg/ml
ULTRA-LEAF purified mouse anti human MHCII (clone: Tü39) Biolegend Custom product based on: RRID: AB_2563139, 10µg/ml
ULTRA-LEAF purified mouse anti MR1(clone: 26.5) Biolegend Cat # 361102; RRID: AB_2562969, 10µg/ml
ULTRA-LEAF purified mouse anti CD1d (clone: 51.1) Biolegend Cat # 350322; RRID: AB_2814281, 10µg/ml
ULTRA-LEAF purified mouse anti IL-12p40(clone: C8.6) Biolegend Cat # 508808; RRID: AB_2810644, 5µg/ml
ULTRA-LEAF purified rat anti IL-18(clone: W17071A) Biolegend Cat # 949704, 5µg/ml

TCR-base stimulation of human cells:
Purified anti human CD3 (clone: OKT3) Biolegend Cat # 317302; RRID: AB_571927, 1.25µg/ml
Purified anti human CD28 (clone: CD28.2) Biolegend Cat # 302902; RRID: AB_314304, 1µg/ml

Flow cytometry mouse cells:
PerCP/Cyanine5.5 anti-mouse/human CD45R/B220 Antibody (clone: RA3-6B2) Biolegend Cat # 103236; RRID: AB_893354 1:200
PerCP/Cyanine5.5 anti-mouse/human CD11b Antibody (clone: M1/70) Biolegend Cat # 101228; RRID: AB_893232 1:200
PerCP/Cyanine5.5 anti-mouse CD11c Antibody (clone: N418) Biolegend Cat # 117328; RRID: AB_2129641 1:200
APC Anti-mouse TCR γ/δ Antibody (clone: GL3) Biolegend Cat # 118116; RRID: AB_1731813 1:200
Alexa Fluor® 700 anti-mouse CD45 Antibody (clone: 30-F11) Biolegend Cat # 103128; RRID: AB_493715 1:300
Brilliant Violet 421™ anti-mouse CD127 (IL-7Rα) Antibody (clone: A7R34) Biolegend Cat # 135023; RRID: AB_10897948 1:100
Brilliant Violet 605™ anti-mouse CD8a Antibody (clone: 53-6.7) Biolegend Cat # 100743; RRID: AB_2561352 1:300
Brilliant Violet 785™ anti-mouse CD4 Antibody (clone: RM4-5) Biolegend Cat # 100552; RRID: AB_2563053 1:200
FITC anti-mouse TCR β chain Antibody (clone: H57-597) Biolegend Cat # 109206; RRID: AB_313429 1:200
Alexa Fluor 488 Anti-human/mouse PLZF Monoclonal Antibody (clone: Mags.21F7) Life Technologies Cat # 53-9320-82; RRID: AB_2574445 1:100
PE/Dazzle™ 594 anti-mouse CD8a Antibody (clone: 53-6.7) Biolegend Cat # 100762; RRID: AB_2564027 1:300
PE/Cyanine7 anti-mouse TCR β chain Antibody (clone: H57-597) Biolegend Cat # 109222; RRID: AB_893625 1:200
APC anti-mouse CD127 (IL-7Rα) Antibody (clone: A7R34) Biolegend Cat # 135012; RRID: AB_1937216 1:100
BV421 Mouse Anti-Mouse RORγt (clone: Q31-378) BD Biosciences Cat # 562894; RRID: AB_2687545 1:200
Brilliant Violet 605™ anti-mouse CD4 Antibody (clone: RM4-5) Biolegend Cat # 100548; RRID: AB_2563054 1:200
Brilliant Violet 605™ anti-mouse CD8a Antibody (clone: 53-6.7) Biolegend Cat # 100744; RRID: AB_2562609 1:300
Brilliant Violet 650™ anti-mouse CD25 Antibody (clone: PC61) Biolegend Cat # 102037; RRID: AB_11125760 1:200
Brilliant Violet 785™ anti-mouse CD45 Antibody (clone: 30-F11) Biolegend Cat # 103149; RRID: AB_2564590 1:300
Brilliant Violet 650™ anti-mouse NK-1.1 Antibody (clone: PK136) Biolegend Cat # 108735; RRID: AB_11147949 1:200
Alexa Fluor® 700 anti-mouse TCR β chain Antibody (clone: H57-597) Biolegend Cat # 109224; RRID: AB_1027648 1:200
PE/Cyanine7 anti-mouse TCR γ/δ Antibody (clone: GL3) Biolegend Cat # 118124; RRID: AB_11204423 1:200
Brilliant Violet 421™ anti-mouse CD8a Antibody (clone: 53-6.7) Biolegend Cat # 100737; RRID: AB_10897101 1:300
Brilliant Violet 605™ anti-mouse CD11c Antibody (clone: N418) Biolegend Cat # 117334, RRID: AB_2562415 1:200
PE CD218a (IL-18Ra) Monoclonal Antibody (clone: P3TUNYA) Life Technologies Cat # 12-5183-80; RRID: AB_2572616 1:100

MHC II blocking in mouse cells:
LEAF™ Purified anti-mouse I-A/I-E Antibody (clone: M5/114.15.2) Biolegend Cat # 107610; RRID: AB_313325 1:100

In vivo depletion of murine CD4 T cells:
InVivoMab anti-mouse CD4 antibody (clone: GK1.5) Bio X Cell Cat # BE0003-1; RRID: AB_1107636 0.5-1mg/mouse titrated per lot

BD Abseq antibodies for Rhapsody single cell sequencing:
CD4, clone: RPA-T4, BD Biosciences Cat # 940304, RRID:AB_2876180, 2µl/test
CD7, clone: M-T701, BD Biosciences Cat # 940029, RRID:AB_2875920, 2µl/test
CD9, clone: M-L13, BD Biosciences Cat # 940078, RRID:AB_2875969, 2µl/test
CD25, clone: M-A251, BD Biosciences Cat # 940463, RRID:AB_2876314, 2µl/test
CD26, clone: M-A261, BD Biosciences Cat # 940101, RRID:AB_2875992, 2µl/test
CD27, clone: M-T271, BD Biosciences Cat # 940018, RRID:AB_2875909, 2µl/test

CD39, clone: TU66, BD Biosciences Cat # 940073, RRID:AB_2875964, 2µl/test
CD45RA, clone: HI100, BD Biosciences Cat # 940011, RRID:AB_2875902, 2µl/test
CD45RO, clone: UCHL1, BD Biosciences Cat # 940022, RRID:AB_2875913, 2µl/test
CD49a, clone: SR84, BD Biosciences Cat # 940094, RRID:AB_2875985, 2µl/test
CD49d, clone: 9F10, BD Biosciences Cat # 940059, RRID:AB_2875950, 2µl/test
CD56, clone: NCAM16.2, BD Biosciences Cat # 940007, RRID:AB_2875898, 2µl/test
CD58, clone: 1C3, BD Biosciences Cat # 940371, RRID:AB_2876240, 2µl/test
CD62L, clone: DREG-56, BD Biosciences Cat # 940041, RRID:AB_2875932, 2µl/test
CD69, clone: FN50, BD Biosciences Cat # 940019, RRID:AB_2875910, 2µl/test
CD72, clone: J4-117, BD Biosciences Cat # 940293, RRID:AB_2876170, 2µl/test
CD73, clone: AD2, BD Biosciences Cat # 940294, RRID:AB_2876171, 2µl/test
CD83, clone: HB15e, BD Biosciences Cat # 940054, RRID:AB_2875945, 2µl/test
CD94, clone: HP-3D9, BD Biosciences Cat # 940081, RRID:AB_2875972, 2µl/test
CD95, clone: DX2, BD Biosciences Cat # 940037, RRID:AB_2875928, 2µl/test
CD103, clone: Ber-ACT8, BD Biosciences Cat # 940067, RRID:AB_2875958, 2µl/test
CD119, clone: GIR-208, BD Biosciences Cat # 940253, RRID:AB_2876134, 2µl/test
CD122, clone: MIK-BETA3, BD Biosciences Cat # 940232, RRID:AB_2876113, 2µl/test
CD123, clone: 7G3, BD Biosciences Cat # 940020, RRID:AB_2875911, 2µl/test
CD124, clone: hIL4R-M57, BD Biosciences Cat # 940092, RRID:AB_2875983, 2µl/test
CD126, clone: M5, BD Biosciences Cat # 940090, RRID:AB_2875981, 2µl/test
CD127, clone: HIL-7R-M21, BD Biosciences Cat # 940012, RRID:AB_2875903, 2µl/test
CD131, clone: 3D7, BD Biosciences Cat # 940385, RRID:AB_2876251, 2µl/test
CD132, clone: TUGh4, BD Biosciences Cat # 940230, RRID:AB_2876111, 2µl/test
CD134, clone: ACT35, BD Biosciences Cat # 940060, RRID:AB_2875951, 2µl/test
CD137, clone: 4B4-1, BD Biosciences Cat # 940055, RRID:AB_2875946, 2µl/test
CD140A, clone:R1, BD Biosciences Cat # 940246, RRID:AB_2876127, 2µl/test
CD140B, clone: 28D4, BD Biosciences Cat # 940237, RRID:AB_2876118, 2µl/test
CD154, clone: TRAP1, BD Biosciences Cat # 940053, RRID:AB_2875944, 2µl/test
CD161, clone: 191B8, BD Biosciences custom product based on Miltenyi: RRID:AB_871628, 2µl/test
CD178, clone: NOK-1, BD Biosciences Cat # 940089, RRID:AB_2875980, 2µl/test
CD181, clone: 5A12, BD Biosciences Cat # 940462, RRID:AB_2876313, 2µl/test
CD183, clone: 1C6/CXCR3, BD Biosciences Cat # 940030, RRID:AB_2875921, 2µl/test
CD192, clone: LS132.1D9, BD Biosciences Cat # 940286, RRID:AB_2876163, 2µl/test
CD196, clone: G034E3, BD Biosciences custom product based on Biolegend: RRID:AB_10918625, 2µl/test
CD197, clone: 3D12, BD Biosciences Cat # 940014, RRID:AB_2875905, 2µl/test
CD212, clone: 2.4E6, BD Biosciences Cat # 940267, RRID:AB_2876146, 2µl/test
CD215, clone: JM7A4, BD Biosciences Cat # 940290, RRID:AB_2876167, 2µl/test
CD278, clone: DX29, BD Biosciences Cat # 940043, RRID:AB_2875934, 2µl/test
CD294, clone: BM16, BD Biosciences Cat # 940098, RRID:AB_2875989, 2µl/test
CD335, clone: 9E2/Nkp46, BD Biosciences Cat # 940064, RRID:AB_2875955 , 2µl/test
GITR, clone: V27-580, BD Biosciences Cat # 940096, RRID:AB_2875987, 2µl/test
IL-21R, clone: 17A12, BD Biosciences Cat # 940099, RRID:AB_2875990, 2µl/test
Itgb7, clone: FIB504, BD Biosciences Cat # 940244, RRID:AB_2876125, 2µl/test
LAG3, clone: T47-530, BD Biosciences Cat # 940080, RRID:AB_2875971, 2µl/test

Validation

All antibodies listed above are commercially available and are validated by their respective manufacturers:
Biolegend (human and mouse) - Flow Cytometry Reagents:
-Specificity testing of 1-3 target cell types with either single- or multi-color analysis (including positive and negative cell types).
-Once specificity is confirmed, each new lot must perform with similar intensity to the in-date reference lot. Brightness (MFI) is evaluated from both positive and negative populations.
-Each lot product is validated by QC testing with a series of titration dilutions.
https://www.biolegend.com/en-us/quality/quality-control

Invitrogen:
Invitrogen antibodies are currently undergoing a rigorous two-part testing approach

Part 1—Target specificity verification
This helps ensure the antibody will bind to the correct target. Our antibodies are being tested using at least one of the following methods to ensure proper functionality in researcher's experiments. Click on each testing method below for detailed testing strategies, workflow examples, and data figure legends.
-Knockout—expression testing using CRISPR-Cas9 cell models
-Knockdown—expression testing using RNAi to knockdown gene of interest
-Independent antibody verification (IAV)—measurement of target expression is performed using two differentially raised antibodies recognizing the same protein target
-Cell treatment—detecting downstream events following cell treatment
-Relative expression—using naturally occurring variable expression to confirm specificity
-Neutralization—functional blocking of protein activity by antibody binding
-Peptide array—using arrays to test reactivity against known protein modifications
-SNAP-ChIP™—using SNAP-ChIP to test reactivity against known protein modifications

-Immunoprecipitation-Mass Spectrometry (IP-MS)—testing using immunoprecipitation followed by mass spectrometry to identify antibody targets

Part 2 -- Specific Application test
https://www.thermofisher.com/uk/en/home/life-science/antibodies/invitrogen-antibody-validation.html

BD:
Antibody specificity
BD Biosciences identifies key targets of interest in scientific research and develops its own specific antibodies or collaborates with top research scientists around the world to license their antibodies. We then transform these antibodies into flow cytometry reagents by conjugating them to a broad portfolio of high-performing dyes, including our vastly popular portfolio of BD Horizon Brilliant™ Dyes.
A world-class team of research scientists helps ensure that these reagents work reliably and consistently for flow cytometry applications.
The specificity is confirmed using multiple methodologies that may include a combination of flow cytometry, immunofluorescence, immunohistochemistry or western blot to test staining on a combination of primary cells, cell lines or transfectant models.
All flow cytometry reagents are titrated on the relevant positive or negative cells. To save time and cell samples for researchers, test size reagents are bottled at an optimal concentration with the best signal-to-noise ratio on relevant models during the product development. To ensure consistent performance from lot-to-lot, each reagent is bottled to match the previous lot MFI. You can look up the Certificate of Analysis and the concentration of test-size human reagents from specific lots via the Concentration Lookup page or BD Regulatory Documents.
Technical data sheets provide data generated on the relevant primary model at this optimal concentration based on a titration curve. QC data on any lot of reagent can be requested through ResearchApplications@bd.com.
Quality control
Our dedication to rigorous testing and high-quality control standards means that you can use our reagents in your research with the utmost confidence. All BD reagent facilities, including our California Design Center at San Diego, our manufacturing facilities in Tatabanya (Hungary) and San Diego (USA), and our California instrument facility (Manufacturing and Design Centers) at San Jose, are approved and registered according to the internationally defined ISO 9001 standard.
Once our research and development (R&D) team completes evaluation of a new product, the developed process is transferred to our manufacturing teams, including Quality Control.
Our manufacturing process adheres to standard operating procedures (SOPs) and guidelines, which are based on ISO requirements and are strictly followed, helping ensure that reagents provide consistent results to help give you assurance of experimental success and confidence in your research.
Quality control testing of newly manufactured lots is performed side-by-side with a previously accepted lot as a control, helping to assure that performance of the new lot is both reliable and consistent.
https://www.bdbiosciences.com/en-us/products/reagents/flow-cytometry-reagents/research-reagents/quality-and-reproducibility

MIltenyi:
With the introduction of recombinant antibodies in 2012, we made a significant investment into improving the quality and consistency of our antibodies. The standardized antibody production process, starting from a defined DNA sequence, and the nature of recombinant antibodies ensure high purity and lot-to-lot consistency.
In addition, recombinant antibodies do not display any undesired mixtures of heavy and light immunoglobulin chains, which is often the case with conventional hybridoma-derived antibodies[5] (PMID: 29485921). Furthermore, our REAfinity™ Recombinant Antibodies have a mutated Fc region that abolishes any binding to Fcγ receptors, resulting in a background-free analysis. These advantages make REAfinity Recombinant Antibodies ideal tools for improving experimental reproducibility.
In 2020, we began the further step of providing antibody validation data directly on our product pages. We do this in order to make it even easier for our customers to choose the antibodies that best match their needs, and to decrease the validation efforts required on their researcher side. With well over 10,000 antibodies in our portfolio, this is an ongoing project and information is updated regularly, so please do check back from time to time. In addition, below you can find some insights on how we conduct our antibody validation process.
https://www.miltenyibiotec.com/US-en/products/macs-antibodies/antibody-validation.html

BioXCell
Advanced Binding Validation: We utilize a library of recombinant proteins and our bioassay expertise to validate that each lot of applicable InVivoPlusTM antibody binds strongly and specifically to its target antigen.
https://bxcell.com/performance-guarantee/

Animals and other organisms

Policy information about studies involving animals; ARRIVE guidelines recommended for reporting animal research

Laboratory animals	C57BL/6J: B6 Charles River, bred in house Strain code: 027<br>B6.129S7-Rag1tm1Mom/J: Rag-/- Charles River, bred in house<br>Il23rgfp/+ : IL-23RGFP Dan Cua (Merck)<br>Cbir1 Charles Elson III<br>HH7-2tg Dan Littman<br>B6.129P2-B2mtm1Unc/DcrJ: B2M-/- Jackson Laboratory Strain code: 002087<br>All animals were age and sex matched between 6 and 12 weeks of age unless indicated otherwise in the age cohort experiment with the exception of the DSS experiment, which was limited to female mice to avoid potential complications from fighting in immunocompromised male mice.
Wild animals	No wild animals were used in the study.

Field-collected samples	No field collected samples were used in the study.
Ethics oversight	Experiments were conducted in accordance with local animal care committees (UK Scientific Procedures Act of 1986) and the Home Department of the Government of the United Kingdom.

Note that full information on the approval of the study protocol must also be provided in the manuscript.

Human research participants

Policy information about studies involving human research participants

Population characteristics	Characteristics of the CRC patients<br>CRC patients n = 49<br>Age (years, average, SD) 68 ± 12<br>Sex (Male/Female) 28/21<br>Time since diagnosis (months, average, SD) 1 ± 4<br><br>Characteristics of the healthy endoscopy patients<br>healthy controls n = 10<br>Age (years, average, SD) 51 ± 9<br>Sex (Male/Female) 7/3<br><br>Characteristics of the UC patients<br>UC patients n = 20<br>Inflamed n = 9<br>Age (Average, SD) 38 ± 19<br>Sex (Male/Female) 4/5<br>Time since diagnosis (years, average, SD) 14 ± 12<br>UCEIS score (average, range) 3.7, [2-6]<br><br>Uninflamed n = 11<br>Age (Average, SD)<br>53 ± 11<br>Sex (Male/Female) 4/7<br>Time since diagnosis (years, average, SD)<br>17 ± 13<br>UCEIS score (average, range) 0.1, [0-1]
Recruitment	Normal adjacent tissue from colorectal cancer (CRC) patients who were undergoing surgery was collected by the TGU biobank. Biopsies from ulcerative colitis (UC) patients were from patients attending the John Radcliffe hospital.
Ethics oversight	All patients gave informed consent and collection was approved by NHS National Research Ethics Service under the research ethics committee (REC) reference 16/YH/0247 (Yorkshire and Humberside REC approval for Translational Gastroenterology Unit GI Illness Biobank, Oxford).

Note that full information on the approval of the study protocol must also be provided in the manuscript.

Flow Cytometry

Plots

Confirm that:

- [x] The axis labels state the marker and fluorochrome used (e.g. CD4-FITC).
- [x] The axis scales are clearly visible. Include numbers along axes only for bottom left plot of group (a 'group' is an analysis of identical markers).
- [x] All plots are contour plots with outliers or pseudocolor plots.
- [x] A numerical value for number of cells or percentage (with statistics) is provided.

Methodology

Sample preparation	Single cell suspensions were isolated from tissues as described in the methods section "Isolation of tissue leukocytes from mouse, Direct digestion of resection-derived tissue, and Generation of single cell suspensions from stored tissue and biopsies" and stained as described in "FACS."
Instrument	Flow cytometry analysis was performed using BD SORP LSRFortessa X20 or BD LSR II. Cell sorts were performed using the FACSAria III.
Software	Collection:<br>FACS Diva Software V 9.1

Analysis:
FlowJo Software V 10.7.1 (BD)
Prism V 9(Graphpad)

Cell population abundance

Because of the low numbers of Tmic in steady-state mice, all cells were sorted for analysis and no post-sort analysis was possible.

Gating strategy

Complete gating strategies are currently omitted from supplementary figures due to space constraints but can be included if required.

☒ Tick this box to confirm that a figure exemplifying the gating strategy is provided in the Supplementary Information.
